# Measuring Disease Activity in Psoriatic Arthritis

**DOI:** 10.1155/2012/839425

**Published:** 2012-12-25

**Authors:** Priscilla C. H. Wong, Ying-Ying Leung, Edmund K. Li, Lai-Shan Tam

**Affiliations:** Department of Medicine and Therapeutics, Prince of Wales Hospital, Chinese University of Hong Kong, Shatin, New Territories, Hong Kong

## Abstract

Over the past decade, the assessment of the disease activity in psoriatic arthritis (PsA) has rapidly evolved in view of the need for valid, feasible, and reliable outcome measures that can be ideally employed in longitudinal cohorts, clinical trials, and clinical practice as well as the growing paradigm of tight disease control and treating to target in the management of PsA. This paper reviews the currently available measures used in the assessment of the disease activity in PsA. The composite measures for PsA that are under development are also discussed.

## 1. Introduction

Psoriaticarthritis(PsA)is a heterogenous multifaceted inflammatory arthritis associated with psoriasis. In addition to peripheral arthritis, patient with PsA may develop spondylitis, dactylitis, enthesitis, and nail disease as well as extra-articular features common to the spondyloarthropathies (SpA). The assessment of disease activity in PsA should therefore evaluate each of these clinical domains carefully. An accurate measurement of disease activity is essential to guide the medical therapy and monitor the treatment response. 

Over the past decade, significant progress on the development and validation of instruments for the measurement of disease activity in PsA has been achieved by the Group for Research and Assessment of Psoriasis and Psoriatic Arthritis (GRAPPA) and the Outcome Measures in Rheumatology Clinical Trials (OMERACT). In 2007, the GRAPPA-OMERACT achieved consensus on 6 core domains that should be included in randomized controlled trials and longitudinal observational cohorts of subjects with PsA [1]. These included peripheral joint activity, skin activity, pain, patient global assessment (PGA), physical function, and health-related quality of life. Several other domains (spinal disease, dactylitis, enthesitis, fatigue, nail disease, radiography, physician global assessment, and acute-phase reactants) were considered important, not mandatory, but preferably to be assessed at some point in a clinical trial development program. This paper reviews the currently available tools for the clinical measurement of the disease activity in PsA. The composite measures for PsA that take different disease domains into account will also be reviewed. 

A collected tools are used in the validation of different patient outcomes measures. These include reliability, validity, and responsiveness. The definition of these tools are first summarized here. In general, assessments of reliability indicate the consistency of responses within a scale or the extent to which a response of score is free from random error (precision). Internal consistency tests the extent that the items of a scale measure the same underlying construct or theme. Intraobserver reliability assesses the likelihood that someone administering a scale to make the same ratings or judgements on repeated administrations. Interobserver reliability assesses the likelihood that two observers to make the same ratings or judgements about the same individual. Validity has been considered to be an expression of the extent to which a question or measure assesses what it is intended to measure. Content validity assesses whether the instrument covers the concepts it is intended to measure. Construct validity assesses whether the measure correlates with measures of other variables in hypothesized ways. Responsiveness or sensitivity to change tests how well the scores on the instrument change to reflect changes in the criterion measure.

## 2. Literature Search

Literature search was conducted using the PubMed database up to November 2012. Different combinations of the following search terms were used: “psoriatic arthritis,” “psoriasis,” “disease activity,” “outcome measures,” “peripheral arthritis,” “dactylitis,” “enthesitis,” “nail,” “spondylitis,” “patient global,” “quality of life,” “fatigue” and “composite measures,” with limits set to include humans and written in English. The initial search yielded more than 3000 abstracts, which were reviewed to include only studies relevant to this paper. This yielded 81 studies, of which the full articles were then reviewed by the authors. 

## 3. Peripheral Joint Assessment

Moll and Wright described five clinical patterns among patients with PsA: distal interphalangeal (DIP), asymmetrical oligoarticular, symmetric polyarticular, spondylitis, and arthritis mutilans [2]. Unlike rheumatoid arthritis (RA), the pattern of joint involvement in PsA is usually asymmetric and frequently involves the DIP joints. Peripheral joints are assessed for tenderness and swelling. There is no validated measure to assess peripheral joint in PsA. The measure used is the American College of Rheumatology (ACR) joint count initially developed in 1949 for the assessment of patients with RA [3]. The ACR joint count ranges from 28, 44, 68, and 78 for tenderness; 28, 44, 66, and 76 for swelling (excluding hips from assessment of swelling). The reduced joint count with the 28 joints or 44 joints count that does not assess the DIP joint or the feet does not have content validity for measuring peripheral arthritis in PsA. Discussions at GRAPPA and OMERACT meetings recommended that the ACR joint count of 68 tender and 66 swollen joints count be used, as it includes a majority of joints affected in PsA, and it can be readily performed in a clinic visit [4]. It was decided not to include the distal joints of the feet (78 tender joints count) as it may be difficult to distinguish proximal interphalangeal (PIP) joint from DIP joint inflammation in the toes. It has been suggested that if either the PIP or DIP of the toe is involved it should be marked as a PIP. The ACR joint count was proven to be a reliable measure of peripheral joint activity in PsA in 3 different studies [5–7]. Gladman et al. showed that the 68 tender and 66 swollen joints count had minimal intraobserver and interobserver variation [6]. The 68 tender and 66 swollen joints count includes the temporomandibular, sternoclavicular, acromioclavicular, shoulder, elbow, wrist (including the carpometacarpal and intercarpal joints as one unit), metacarpophalangeal (MCP), PIP, DIP, hip, knee, talotibial, midtarsal (including subtalar), metatarsophalangeal, and interphalangeal joints of the toes (proximal and distal joints of each toe counted as one unit). 

## 4. Skin Assessment

PsA disease activity is commonly not mirrored by active skin disease. A wide variety of scoring systems have been proposed to evaluate severity in psoriasis. A systemic review of all clinical studies (prospective and retrospective) investigating the severity of psoriatic patients was published in 2010 [8]. Based on methodological validation and quality criteria, six clinical severity scores were selected and analyzed (Table 1). They included Body Surface Area (BSA) [9], Psoriasis Area, and Severity Index (PASI) [10], the Physician's Global Assessment (PGA) [11], the Lattice System Physician's Global Assessment (LS-PGA) [11], the Self-Administered PASI (SAPASI) [12], and the Salford Psoriasis Index (SPI) [13]. It appeared that none of the severity scores used for psoriasis met all of the validation criteria required for an ideal score. However, it was concluded that the PASI score was the most extensively studied psoriasis clinical severity score and the most thoroughly validated. PASI was demonstrated to be reliable and reproducible. Its internal consistency [11], intraobserver reliability [11, 12, 14] and interobserver reliability [11, 13, 14] are good and its sensitivity to change is acceptable [12]. It has a good content validity. Evidence-based recommendations to assess psoriasis severity stated that the PASI can be used in everyday clinical practice in the management of adult patients with plaque-type psoriasis, in particular, if a systemic treatment is considered [15]. However, PASI has a number of drawbacks. Its construct validity and its acceptability have not been evaluated. It has poor sensitivity to change and responsiveness when skin psoriasis is less than 10% BSA involvement. The correlation with quality of life measures is poor [16]. Spuls et al. therefore suggested drawing on multiple measurement tools to fully characterize disease severity and responsiveness to therapy [17]. The PASI and LS-PGA, for example, complement each other and provide a representative picture of disease severity [17].

## 5. Dactylitis

Dactylitis is characterized by swelling of a whole digit and represents a combination of synovitis and inflammation of tendon and ligament insertions. It is a hallmark feature of PsA, occurring in 16–48% of reported cases [18]. It can be further characterized as acute dactylitis where the digit is erythematous, swollen, hot, and tender to touch; or as chronic dactylitis where the digit is swollen but without redness and tenderness. There is a study that demonstrated that digits with dactylitis are associated with a greater degree of radiological damage than those which occur in digits not affected by dactylitis [19]. 

The Leeds Dactylitis Instrument (LDI) was developed in response to the need for a clinical, objective, validated outcome measure for dactylitis [18]. It measures the ratio of the circumference of the affected digit to the circumference of the digit on the contra-lateral hand or foot: a minimum difference of 10% is used to define a dactylitic digit. If the contralateral digit is also dactylitic, a table of normative values based on population averages is used to provide the comparison. The ratio of circumference is multiplied by a tenderness score, originally based on the Ritchie index (graded 0–3), but a later modification amended this to a binary score (0 for nontender, 1 for tender—this later modification is referred to as the LDI basic). The results from each digit with dactylitis are then summed to produce a final score. The aim of the LDI is to provide a quantification of both the size of the swollen digit and the tenderness so that the score can differentiate between tender and nontender dactylitis. Both the LDI and LDI basics have demonstrated good inter- and intraobserver reliability [18]. The LDI was tested in the International Multicenter Psoriasis and Psoriatic Arthritis Reliability Trial (IMPART) and showed good agreement among rheumatologists but not dermatologists [20].

## 6. Enthesitis

Enthesitis is defined as inflammation at the site of tendons, ligaments, or joint capsule fibre insertion into bone. It is a unique and important clinical feature of spondyloarthropathy. Most clinical studies have reported the frequency of enthesitis in PsA cohorts in the 30–50% range [21, 22], but this may be an underestimate, as now imaging studies such as ultrasonography or magnetic resonance imaging (MRI) have demonstrated enthesopathy not appreciated clinically. 

**Figure 1:**
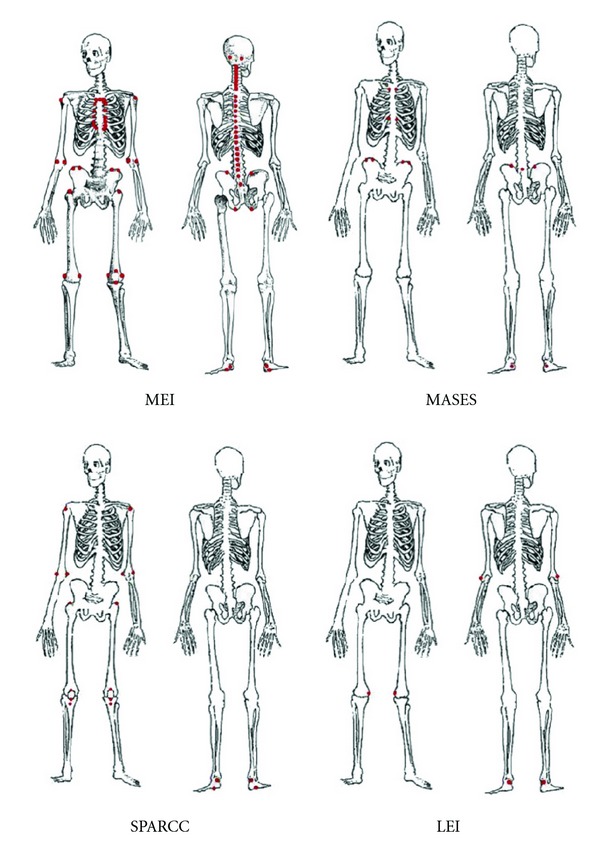
Enthesitis sites recorded by the principle enthesitis indices [32]. MEI: Mander Enthesitis Index; MASES: Maastricht Ankylosing Spondylitis Enthesitis Score; SPARCC: Spondyloarthritis Research Consortium of Canada Enthesitis Index; LEI: Leeds Enthesitis Index.

In 1987, Mander et al. published the first instrument to investigate enthesitis in ankylosing spondylitis (AS), the Mander enthesis index (MEI) [23]. 66 entheseal sites which were accessible to clinical examination were defined (Figure 1). These sites were to be examined for tenderness and the intensity of pain was graded on a 0 to 3 scale. The MEI is sensitive to change in clinical state associated with nonsteroidal antirheumatic drug therapy, and one study demonstrated good intraobserver reliability [24]. On the other hand it was not possible to demonstrate any treatment group differences in a large placebo-controlled trial of infliximab in AS using the MEI, suggesting that discrimination was poor because of low interobserver reliability [25]. MEI is generally regarded as being nonfeasible in clinical use because it is time consuming and not all enthesitic sites are readily identifiable on physical examination. It has also been criticized for potentially causing distress to patients, and not adequately distinguishing enthesitic sites from fibromyalgia tender points. 

The Maastricht Ankylosing Spondylitis Entheses Score (MASES) was developed to modify MEI to produce a less time consuming index with similar validity [26]. The grading of tenderness score from 0 to 3 in MEI was removed and substituted with a dichotomous 0/1 score for tenderness in MASES. The number of entheses index was reduced as concise as possible. After exclusion of entheses difficult to localize or near to other sites, the 66 entheses were reduced to 13 most specific and sensitive sites. These include the bilateral first and seventh costochondral joints, the anterior and posterior superior iliac spines, the iliac crests, the fifth lumbar spinous process, and the proximal insertion of Achilles tendon (Figure 1). MASES is a more feasible instrument but it has not been assessed in other diseases of the SpA including PsA. The fact that it does not score one of the main enthesitis sites (plantar fascia insertions into the calcaneum) also gives rise to some concern. 

Gladman et al. have reported on the performance of investigators from the Canadian Spondyloarthropathy Group in their ability to reliably assess four enthesitis areas: rotator cuff insertion at the shoulder, tibial tuberosity at the knee, Achilles tendon, and plantar fascia insertions in the calcaneus [6]. The reliability was “fair” in the assessment of rotator cuff enthesitis, “moderate” for tibial tuberosity and Achilles enthesitis, and “moderate to substantial” for plantar enthesitis. This may have been due to difficulties in the anatomic localization of rotator cuff entheses. 

In a study on infliximab in patients with AS, Braun et al. used an enthesis index composed of 12 entheses that are reported to be commonly affected in the inflammatory process in AS (major enthesitis index) [27]. This index included the iliac crests, the great trochanters of the femur, the medial and lateral condyles of the humerus, the proximal insertion of the Achilles tendon, and insertion of the plantar fascia to the calcaneus. This index did not perform better compared to the MASES in the above-mentioned study. It has not been widely used or studied. 

The Spondyloarthritis Research Consortium of Canada (SPARCC) created a new outcome measure for enthesitis in SpA using information from ultrasound and MRI studies [28]. The selection of entheses was based on two published findings from power Doppler ultrasound and MRI [29, 30]. Sixteen sites were selected: the bilateral greater trochanter, quadriceps tendon insertion into the patella, patellar ligament insertion into the patella and tibial tuberosity, Achilles tendon insertion, plantar fascia insertion, medial and lateral epicondyles, and the supraspinatus insertion (Figure 1). Tenderness at each site was quantified on a dichotomous basis: 0 means nontender and 1 means tender. Interobserver reliability was good and a substantial correlation was seen between the total enthesitis score and other disease activity measures [28]. 

The MEI, the MASES, the Gladman index, and the major enthesitis index were all developed and validated for patients with AS. The SPARCC score was developed using a full spectrum of SpA patients, but the validation was only done in patients with AS [28]. The Leeds Enthesitis Index (LEI) which was published in 2008 is the only measure developed specifically for PsA [31]. The LEI consisted of 6 sites: bilateral Achilles tendon insertions, medial femoral condyles, and lateral epicondyles of the humerus (Figure 1). Tenderness at each site was quantified on a dichotomous basis: 0 means nontender and 1 means tender. This index was compared with other entheseal indices including the MEI, MASES, the Gladman index and the major enthesitis index in an open-label longitudinal study. The LEI was able to distinguish between patients with active disease and those without. It showed strong correlation with other disease activity measures, a large effect size, and the lowest floor effect. Floor effect means not picking up cases with low index score. A low floor effect means that it will be likely to detect the large majority of patients with active enthesitis. 

The reproducibility of enthesitis assessment among patients with PsA with spinal involvement was investigated in the International Spondyloarthritis Interobserver Reliability Exercise—the INSPIRE study [7]. Enthesitis sites included in the MASES, SPARCC, LEI, and other enthesitis scoring systems were investigated. The results showed that there was excellent agreement among the observers with regard to the number of active enthesitis sites per individual patient. The individual indices provided substantial to excellent agreement for all patients. The LEI performs well in PsA. 

## 7. Nail Assessment

Nail involvement is common in patients with psoriasis and PsA and can be severe and disfiguring. Nail psoriasis occurs in as many as 50% of psoriatic patients [33] and has been reported from 63% to 83% in patients with PsA [34, 35]. 

Psoriatic nail disease can be broadly divided into psoriasis affecting the nail matrix and the nail bed. Involvement in the nail matrix results in changes to the nail plate. Characteristic features of psoriasis affecting the nail matrix include pitting, leukonychia, red spots in the lunula, and nail plate crumbling. Characteristic features of psoriasis affecting the nail bed include oil-drop discolouration, onycholysis, nail bed hyperkeratosis, and splinter hemorrhages. 

In 2003, a group of dermatologist had developed the Nail Psoriasis Severity Index (NAPSI) as a psoriatic nail grading instrument [36]. This instrument is used to evaluate the severity of nail matrix psoriasis and nail bed psoriasis by area of involvement in the nail unit. The nail is divided with imaginary horizontal and longitudinal lines into four quadrants. Each quadrant of the nail is evaluated by presence of any of the nail matrix and nail bed psoriasis features. The score range from 0 to 4 according to the number of quadrants with any of the features present. Each nail gets a nail matrix score (0–4) and a nail bed score (0–4), and the total nail score is the sum of these two (0–8). The sum of the scores from all nails is 0–80 or 0–160 if toenails are included. If a target nail scale is desired, the same technique can be used to evaluate all eight parameters (pitting, leukonychia, red spots in lunula, crumbling, oil drop, onycholysis, hyperkeratosis, and splinter hemorrhages) in each quadrant of the nail, giving that one nail scores 0–32. An informal survey of the NAPSI score suggested that the NAPSI score was reproducible among dermatologists grading the same nails. However, the NAPSI scores was not validated. 

In 2007, a group of rheumatologists with a goal to validate a psoriatic nail disease assessment instrument to assess disease severity and response to treatment in clinical trials had modified the original NAPSI in an attempt to enhance its reliability and face validity [37]. First, the division of the nail into quadrants was eliminated because quadrants were felt to be difficult to precisely quantify and varied among observers. Second, a more quantitative aspect was added to the scoring of several features in order to increase the sensitivity of the overall grading. Nail pitting was scored 0–3 depending on the number of pits present. Crumbling and onycholysis were scored 0–3 depending on the percentage of the nail involved. Splinter hemorrhages, leukonychia, red spots in the lunula, oil-drop dyschromia, and nail bed hyperkeratosis were individually scored as 1 if they were present and 0 if they were absent. Oil-drop dyschromia was regarded as part of the same pathologic process as onycholysis, and therefore oil-drop dyschromia and onycholysis were graded together. In the end, the range of possible scores using the modified NAPSI (mNAPSI) was 0–14 for each fingernail, or 0–140 for all 10 fingernails. In addition to the mNAPSI, global nail psoriasis severity ratings from both patients and physicians were added using a visual analogue scale (VAS) (0–10). The mNAPSI scores showed excellent internal consistency and interobserver reliability [37]. Its construct validity was shown by the correlation between mNAPSI scores and global nail severity VAS scores and by correlation between the physician and patient global assessments of nail disease activity.

## 8. Spine Assessment

Spondylitis has been reported in 40–51% of PsA patients [38]. Sacroiliitis has been reported in up to a quarter of patients in several series [39–41]. Unlike AS, where axial involvement is present in all patients and tends to be more severe both clinically and radiologically, axial PsA is more heterogenous and less severe than that in AS. Up till now, there is no consensus on the definition of axial PsA. The assessment for spinal involvement has been borrowed from the Assessment of Ankylosing Spondylitis (ASAS) working group. The ASAS working group has recommended the Bath Ankylosing Spondylitis Disease Activity Index (BASDAI) to measure the disease activity [42], the Bath Ankylosing Spondylitis Function Index (BASFI) to evaluate the functional ability [43], and the Bath Ankylosing Spondylitis Metrology Index (BAMI) to assess spinal mobility [44]. The International Spondyloarthritis Interobserver Reliability Exercise (INSPIRE) study showed that the axial measures of spinal mobility used in AS perform well with respect to interobserver reliability and are equally reproducible when applied to PsA patients with axial involvement [45]. There are studies showing BASDAI correlated highly with patient perception of disease activity but there was no significant effect of the pattern of disease (axial or peripheral) [46, 47], suggesting that the BASDAI does not differentiate between axial and peripheral disease activity. 

## 9. Patient Global Assessment

PGA of disease activity is important because it enhances the patient physician interaction to become more patient-centered by highlighting the global influences of PsA on the individual patient's well-being. The PGA is very dependent on the wording of the question poses to patient. In PsA, when the patients are being evaluated for PGA, they (and even the clinicians) may get confused whether they should relate the assessment to joint involvement, or skin involvement, or both. To address this issue, the GRAPPA organized a multicenter study to assess the reliability of the PGA, measured by means of 0–100 mm VAS, and the additional utility of separate VAS scales for joints (PJA) and skin (PSA) [48]. The specific question for PGA was “In all the ways in which your psoriasis and arthritis, as a whole, affect you, how would you rate the way you felt over the past week?” Results showed that PGA with a single question addressing both joint and skin disease is a reliable and responsive measure in assessing patient in totality. Because joint and skin disease often diverges, it is suggested that in some circumstances, such as study of a drug that improves the joints but not the skin, both PJA and PSA are also assessed.

## 10. Health-Related Quality of Life

The most commonly used measure of health-related quality of life include the Health Assessment Questionnaire (HAQ), the Medical Outcomes Study Short Form 36 (SF-36), the Psoriatic Arthritis Quality of Life (PsAQoL), Dermatology Life Quality Index (DLQI), and the EuroQol 5-domain (EQ-5D). 

The HAQ was originally developed for the assessment of disability in patients with RA [49]. It focuses on 2 dimensions of health status, physical disability (8 categories), and pain. The 8 categories, reviewing a total of 20 specific functions, evaluate patient's difficulty with activities of daily living over the past week. The 8 categories include dressing and grooming, arising, eating, walking, hygiene, reaching, gripping, and errands and chores. It also identifies specific aids or devices utilized for assistance, as well as help needed from another person. The HAQ has been modified for spondyloarthropathies (HAQ-S), which includes 2 spinal domains (SPAR1 and SPAR2) [50]. It has also been further modified for psoriasis (HAQ-SK) [51]. Both the HAQ-S and HAQ-SK scores were shown to perform similarly to the original HAQ score [52], suggesting that neither the spinen or the skin related questions enhance the assessment of health status provided by the original HAQ. 

The SF-36 is a generic health assessment questionnaire intended to measure general health concepts not specific to any age, disease, or treatment group [53]. It measures 8 health domains: physical functioning, pain, vitality, social functioning, psychological functioning, general health perceptions, and role limitations due to physical and emotional problems. It also can be subdivided into two summary scores, the physical component summary and the mental component summary scores. This instrument has been validated in PsA [54]. It was found to be reliable in patients with PsA and could be used to distinguish PsA patients from the general population.

PsAQoL is a 20-item, PsA-specific health-related QOL instrument. It has shown reliability and construct validity, but its use in clinical trial has not yet been published [55].

DLQI is a 10-item questionnaire developed as a measure of disability for a wide range of dermatological conditions [56]. It has been validated in assessment of psoriasis and shows discrimination and responsiveness in PsA trials [57–59]. 

The EQ-5D is comprised of a 5-dimension set of health status measures and a VAS [60]. The 5 dimensions are mobility, self-care, usual activities, pain/discomfort, and anxiety/depression. Each dimension has 3 levels: no problems, some problems, and extreme problems. The VAS records the respondent's self-rated health on a 20-centimeter vertical VAS where the endpoints are labelled “the best imaginable health state” and “the worst imaginable health status.” The EQ-5D has shown discrimination and responsiveness in PsA trials [60].

Currently, there is no single generally agreed upon definition or conceptual model of health-related quality of life. The choice of the different measures of health-related quality of life in PsA depends on its content, respondent burden, administrative burden, translation and adaptations, acceptability, reliability, validity, and ability to detect change. PsAQoL is the only measure specific to PsA. It is now being used for randomized controlled trial, from which more will be learned about its performance characteristics. 

## 11. Fatigue

Fatigue in varying degrees is a frequent, often debilitating problem that significantly affects patients' lives. Fatigue can be constant and persistent, or fluctuating and unpredictable. Several scales have been used to assess fatigue in rheumatic diseases, including the Fatigue Severity Scale (FSS) [61], the Functional Assessment of Chronic Illness Therapy-Fatigue (FACIT-F) [62], the Multidimensional Assessment of Fatigue (MAF) [63], the Multidimensional Fatigue Inventory (MFI) [64], the Profile of Fatigue (ProfF) [65], the Short Form 36 Vitality Subscale (SF-36 VT) [53] and the Visual Analog Scales for Fatigue [66]. A modified version of the FSS has been validated in PsA [67]. The FACIT-F was validated in a Toronto PsA cohort study. It correlated well with the modified FSS, showing high internal consistency, test-retest reliability, criterion, and construct validity [68].

Currently no single measure is favoured in use for PsA patients. The area of fatigue measure in PsA still needs to be further studied.

## 12. Composite Measures

A composite measure is a one way of assessing all relevant clinical outcomes in a single instrument. It incorporates several dimensions of disease status, often by combining these different domains into a single score. Given with the large number of domains that may be affected in a single patient with PsA, developing a comprehensive composite measure is a major challenge. The GRAPPA and the society OMERACT are working on the development of composite measures of disease severity and responses to therapy that take into account most of the disease domains. 

Disease Activity for Psoriatic Arthritis (DAPSA) was adapted from the Disease Activity Index for Reactive Arthritis (DAREA) [69], a score developed and validated for reactive arthritis. DAPSA was developed from a clinical cohort [70] and validated using clinical trial data [71]. It comprises 68 tender and 66 swollen joints count, patient global, pain, and C-reactive protein level (Table 2). The composite score is a simple sum of the scores. Skin assessment was excluded because it did not reach statistical significance. 

The Psoriatic Arthritis Joint Activity Index (PsAJAI) was developed from pooled data from randomized clinical trials of antitumor necrosis factor agents in PsA [72, 73]. A response is defined as a 30%-improvement in six measures with weights of 2 given to tender and swollen joint counts, C-reactive protein, and physician global assessment of disease activity (Table 2). Weights of 1 are given to pain, patient global assessment of disease activity, and the HAQ. 

Composite Psoriatic Disease Activity Index (CPDAI) was a domain-based measure [74]. Disease involvement is assessed in up to 5 domains: peripheral arthritis, skin, enthesitis, dactylitis, and spinal manifestations (Table 2). For each domain, instruments are used to assess both the extent of disease activity and the effect of involvement in that domain on patients' function and health-related quality of life. Domains are scored from 0 to 3, with empirical cutoffs for disease severity/activity proposed in each one, largely based on the literature. Individual domain scores are summed to give an overall composite score (range 0–15) [74]. 

The CPDAI has been validated in a large clinical trial dataset Psoriasis Randomized Etanercept Study in Subjects with Psoriatic Arthritis (PRESTA) [75]. In PRESTA, 752 patients were randomized to a double-blind, 2-period study that evaluated the safety and efficacy of 2 doses of etanercept on skin and musculoskeletal disease. Joint responses were equally determined by both the CPDAI and the DAPSA composite scores; but it was only the CPDAI, which also encompasses other domains including skin, enthesitis, and dactylitis, being able to discern the global treatment response between the 2 etanercept doses. This demonstrates that the CPDAI is a more sensitive instrument to detect changes in the different domains of disease activity in PsA. 

## 13. GRACE Project 

In an attempt to develop new composite outcome measures for PsA, the GRAppa Composite Exercise (GRACE) project has been developed following the GRAPPA annual meeting in 2008. This is a long-term project with longitudinal observational data which are being collected on a large cohort of PsA patients at multiple centres internationally. To date, baseline information on 503 patients with PsA has been collected [76, 77]. Recently 2 novel indices, Psoriatic Arthritis Disease Activity Score (PASDAS) and Arithmetic Mean of Desirability Functions (AMDF) were developed using multiple linear regression and physician-defined cutoffs for disease activity, respectively [76]. It is anticipated that further testing in other datasets including comparison with existing measures will be done to validate these new instruments.

## 14. Minimal Disease Activity 

Minimal disease activity is a concept that has been defined by the OMERACT as a state of disease activity deemed a useful target of treatment by both the patient and physician. Coates et al. had developed minimal disease activity (MDA) criteria for PsA using data on 40 patients with the disease and the expert opinions of 60 rheumatologists and dermatologists [78]. The goal of the development of this instrument is to “treat to target,” to achieve disease remission or low disease activity state. For a patient to achieve MDA, 5 of the 7 criteria must be met (Table 3). The MDA criteria has been validated in an observational cohort and 2 infliximab studies of PsA [79, 80]. They showed patients with active PsA achieving MDA with effective therapy having a significant reduction in joint damage radiographic progression. 

## 15. Conclusion

Given with the complexities of the disease nature of PsA, assessment of disease activity needs to take into account the core set of domains in order to assess their impact on the patient and the response to treatment. There are still considerable controversial issues about the content and performance of composite measures. Efforts by the OMERACT/GRAPPA working group are underway to determine if composite measures that capture both disease activity and response to therapy can be developed that effectively encompass all of the domains. Currently there is great heterogeneity in PsA assessment, even since publication of the OMERACT core set. Better consensus on instruments to assess each domain of disease activity in PsA is anticipated.

## Figures and Tables

**Table 1:** Clinical assessment of psoriasis outcome measures.

	Erythema	Induration	Desquamation	BSA	Psychosocial impact	Measured by
BSA				Yes		Physician
PASI	Yes	Yes	Yes	Yes		Physician
PGA	Yes	Yes	Yes			Physician
LSPGA	Yes	Yes	Yes	Yes		Physician
SAPASI	Yes	Yes	Yes	Yes		Patient
SPI	Yes	Yes	Yes	Yes	Yes	Physician

BSA: Body Surface Area; PASI: Psoriasis Area and Severity Index; PGA: Physician's Global Assessment; LGPGA: Lattice System Physician's Global Assessment; SAPASI: Self-Administered PASI; SPI: Salford Psoriasis Index.

**Table 2:** Clinical domains included in composite measures in PsA.

	Peripheral arthritis	Pain	Patient global assessment	Physician global assessment	Skin	Enthesitis	Dactylitis	Spine disease	HAQ	CRP
DAPSA	Yes	Yes	Yes							Yes
PsAJAI	Yes	Yes	Yes	Yes					Yes	Yes
CPDAI	Yes		Yes		Yes	Yes	Yes	Yes	Yes	

DAPSA: Disease Activity for Psoriatic Arthritis; PsAJAI: Psoriatic Arthritis Joint Activity Index; CPDAI: Composite Psoriatic Disease Activity Index; HAQ: Health Assessment Questionnaire; CRP: C-reactive protein.

**Table 3:** Minimal disease activity (MDA) criteria in psoriatic arthritis.

A patient is classified as in MDA when 5 of the following 7 criteria are met:	
Tender joint count ≤1	
Swollen joint count ≤1	
PASI ≤1 or BSA ≤3	
Patient pain VAS ≤15	
Patient global activity VAS ≤20	
HAQ ≤0.5	
Tender enthesial points ≤1	

PASI: Psoriasis Area and Severity Index; BSA: Body Surface Area; VAS: Visual Analog Scale; HAQ: Health Assessment Questionnaire.
